# Anti-tuberculous Effects of Statin Therapy: A Review of Literature

**DOI:** 10.7759/cureus.7404

**Published:** 2020-03-25

**Authors:** Faryal Tahir, Taha Bin Arif, Jawad Ahmed, Syed Raza Shah, Muhammad Khalid

**Affiliations:** 1 Internal Medicine, Dow University of Health Sciences, Karachi, PAK; 2 Cardiology, Kansas City University of Medicine and Biosciences, Joplin, USA; 3 Cardiology, Ascension Via Christi Hospital, Pittsburg, USA

**Keywords:** statins, tuberculosis, tb, mycobacterium tuberculosis, hmg-coa reductase inhibitors

## Abstract

Tuberculosis (TB) is a chronic infection caused by *Mycobacterium tuberculosis* (M. TB). It is transmitted through respiratory droplets. Increased cholesterol level is a predisposing factor for TB. M. TB uses cholesterol in the host macrophage membranes to bind and enter the macrophages. Statins are the drugs that are prescribed to hyperlipidemic patients to maintain their lipid levels in the normal range, thereby reducing the risk of stroke and cardiovascular events. Moreover, statins aid in reducing the levels of cholesterol in human macrophages. Therefore, a reduction in the membrane cholesterol minimizes the entry of TB pathogen inside macrophages. Furthermore, acting as vitamin D3 analogs and positively influencing pancreatic beta-cell function in a chronic diabetic state, statins minimize the occurrence of M. TB infection among diabetic population as well. This review aims to provide a comprehensive detail of all in vitro, in vivo, and retrospective studies that investigated the effects of statins in relation to the prevention or treatment of TB infection.

## Introduction and background

Tuberculosis (TB) is a chronic infection caused by *Mycobacterium tuberculosis* (M. TB). In 2018, it was estimated that nearly 10 million people got infected with TB. It is one of the top 10 causes of death worldwide, even surpassing human immunodeficiency virus/acquired immunodeficiency syndrome (HIV/AIDS). A total of 1.5 million deaths were reported from TB in 2018. Pakistan was among the eight countries that were responsible for two-thirds of the TB cases reported worldwide. Globally, the rate of TB infection is falling by 2% annually, and it has been estimated that nearly 58 million lives have been saved between 2000 to 2018 via adequate diagnosis and treatment of TB [1]. M. TB is transmitted through respiratory droplets. They may enter the respiratory tract causing pulmonary TB or may disseminate and spread to other areas of the body such as the abdomen, lymph nodes, bones, meninges, and spine. The droplets containing the bacteria can cross the mucociliary barrier, but the bacteria are immediately surrounded and engulfed by macrophages [2]. The host body mediates an immune response via CD4+ and CD8+ T cells along with the formation of antibodies against M. TB. In an immunocompetent individual, these responses can contain the bacteria and stop its progression but the bacteria can evade detection and elimination via macrophages and persist in the body. However, this latent infection may get reactivated when the body’s immune system is weakened [3]. Conditions like diabetes and increased cholesterol in the body make an individual more prone to TB infection [4,5].

Statins are the drugs that are prescribed to hyperlipidemic patients to maintain lipids levels in the normal range, thereby reducing the risk of stroke and cardiovascular events. They work by blocking 3-hydroxy-3-methylglutaryl coenzyme A (HMG CoA) reductase, the rate-limiting enzyme in the cholesterol synthesis pathway [5-6]. In his review, Stancu C *et al*. have explained the mechanism of action of statins in detail [6]. They act as competitive agonists that bind to the active site of HMG CoA reductase enzyme and prevent the binding of the actual substrate (HMG CoA). This leads to the decreased synthesis of cholesterol and reduced cholesterol levels in the hepatocytes. Sterol regulatory element-binding protein (SREBP) is cleaved from the endoplasmic reticulum (ER) and it translocates into the nucleus. SREBP binds to the sterol regulatory element (SRE) in the nucleus. This causes the formation of low-density lipoprotein receptor (LDL-R) gene, which, in turn, is transcribed to LDL-R messenger RNA (mRNA). Translation of LDL-R mRNA causes the synthesis of LDL-Rs, which mature and travel to the surface of hepatocytes. Freely circulating low-density lipoproteins (LDL) bind to LDL-R on hepatocytes and move in the liver cells to maintain intracellular cholesterol levels. This increased movement of LDL from the circulation into the hepatocytes causes a reduction in serum LDL (bad cholesterol) levels [6]. Statins are also known to increase the levels of good cholesterol i.e. high-density lipoproteins (HDL). Notable side effects of statins include, but are not limited to, myalgia, hepatotoxicity, flushes, digestive tract problems, and rashes. A few examples of statins include fluvastatin, pitavastatin, atorvastatin, simvastatin, lovastatin, rosuvastatin, and pravastatin [6].

Increased cholesterol levels is a predisposing factor for TB. M. TB uses cholesterol in the host macrophage membrane to bind and enter the macrophage [7-8]. M. TB also inhibits phagosomal activation and can persist in the inactivated (pathogen-friendly) phagosome without being lysed [9]. A reduction in membrane cholesterol decreases the entry of TB pathogen inside macrophages [8]. Retrospective human studies and experimental animal studies have shown beneficial effects of statins in both treatment and prevention of TB [10-12]. Classic first-line TB treatment consists of four drugs: rifampicin, isoniazid, pyrazinamide, and ethambutol. Statins, as an adjunct, have also been found to increase the effects of first-line TB drugs [13].

No randomized controlled trial (RCT) has been carried out to study risk versus benefit of statins for TB. However, several new in vitro and in vivo studies have been published in the last few years. This review aims to provide comprehensive detail of all in vitro, in vivo, and retrospective studies that reported the effects of statins in relation to the prevention or treatment of TB infection.

## Review

Why mycobacterium has a longer life-span inside macrophage?

The cycle of TB infection begins with the dispersion of M. TB aerosols. There is a more likely risk of transmission when approximately one to 10 bacilli are dispersed through the air. M. TB, being a potent facultative intracellular pathogen, eludes host immunity and is taken up by the alveolar macrophages via phagocytosis [5]. Phagocytosis is one of the major mechanisms of innate immunity and is among the initial processes responding to infection [5]. The cells that are responsible for phagocytosis are called phagocytes which include neutrophils, macrophages, monocytes, dendritic cells, osteoclasts, and eosinophils. Among them, macrophages act as the first line of defense against various intracellular pathogens [14]. When M. TB enters the macrophage, it becomes confined in a membrane-bound vesicle, known as phagosome [5]. M. TB, a highly successful bacillus, has developed numerous strategies to overcome critical innate-effector immune responses of macrophages such as fusion of phagosome with the lysosome, presentation of the antigen, autophagy, and inhibition of reactive oxygen and nitrogen species (ROS and RNS) to ensure its longer survival inside the macrophage [3,15-16]. For instance, this phagosome maturation arrest increases the life span of the respective macrophages [5]. Another factor contributing to this maturation arrest is the inhibition of the fusion of vesicles between stages controlled by rab5 and rab7 [17]. Moreover, immunological response via T lymphocytes is also modulated which further aids prolonged residence of bacilli within the macrophage [18]. This is the general principle behind the long-standing disease that leads to progression in the case of TB infection inside human hosts. The condition is supersaturated by the fact that inducible nitric oxide synthase (iNOS) is found to be comparatively less co-localized with the phagosome accommodating mycobacterium [19]. Dissociation of the phagosome from its coat protein, i.e. Tryptophan aspartate-containing coat (TACO) protein and the generation of cyclical waves of phosphatidylinositol 3-phosphate (PI3P) inside the macrophage is vital for the maturation of phagosome, both of which are hampered in case of active mycobacterial infection [5,19-20].

What is the role of cholesterol in the development of TB infection?

Cholesterol in the membrane of host macrophage has been regarded as a high-affinity docking site responsible for stable anchoring of M. TB pathogen prior to phagocytosis [7]. M. TB exhibits cholesterol-binding protein that attaches to the cholesterol-enriched domains of the host macrophage [21]. Hence, M. TB enters the macrophage when there is an ample amount of cholesterol which allows its binding. For instance, depletion of membrane cholesterol has been proved to inhibit the introduction of TB pathogen inside host macrophages [8].

The formation of a phagosome following the successful entry of M. TB pathogen inside macrophage is influenced by the amount of cholesterol in the plasma membrane. The premature formation of PI3P and its binding with Rab5 and other binding proteins occurs in a cholesterol dependant manner. The persistent nature of M. TB relies on its ability to inhibit phagosomal maturation. It resides inside a pathogen-friendly phagosome escaping lysosomal fusion, an event critical for bactericidal mechanisms. Following the entry via cholesterol-rich domains, mycobacteria are sequestered in the phagosomes [5]. Upon phosphorylation, nicotinamide adenine dinucleotide phosphate hydrogen (NADPH) oxidase is activated and TACO protein is dissociated from the phagosome [5]. For the clearance of bacteria, this maturation process is vital for the fusion of pathogen containing phagosome with the lysosome. But, in case of M. TB, phagosome maturation arrest has been observed as there is less effective phosphorylation of NADPH oxidase subunits and TACO protein [4]. Furthermore, due to the accumulation of cholesterol, non-dissociation of Rab7 protein from tubercle bacilli containing phagosome seems to be another factor responsible for maturation arrest [9].

The engagement of NADPH oxidase subunits with the phagosome is believed to be cholesterol-dependent [5]. Hence, cholesterol may be vital for the regulation of enzyme activity [22-23]. Moreover, TACO occupies the cholesterol-enriched zones of the leukocytes; therefore, its association with the phagosome also seems to be dependent on the amount of cholesterol in the phagosomal membrane [24]. So, the more abundant the cholesterol in the membrane, the longer the survival of M. TB pathogen inside TACO-shielded stable phagosome. In addition to this, increased amounts of membrane-associated Rab7 due to the accumulation of cholesterol causes loss of dynamic properties including motility of late endocytic vesicles, resulting in the failure of phagosomal fusion with the lysosome [25].

A sterol metabolism, utilizing host cholesterol, has been observed as a crucial step towards the development of M. TB infection [26-27]. Thus, the host cholesterol plays an essential role in ensuring the persistence of long-standing tubercle bacilli [28]. Moreover, the longer life span of intracellular tubercle bacilli is aided by oxidized LDL laden macrophages [29]. For instance, a study proposed this cholesterol catabolism as a therapeutic target to control the progress of M. TB infection [30].

How does statin therapy prevent TB infection?

**Figure 1:**
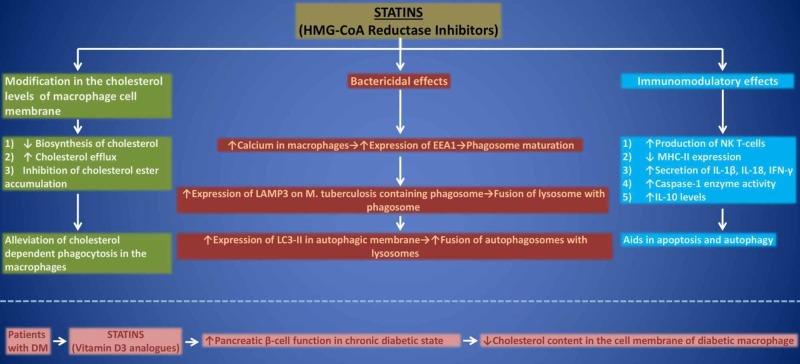
Anti-tuberculous effects of statin therapy HMG-CoA, 3-hydroxy-3-methylglutaryl coenzyme A; EEA1, early endosomal antigen 1; LAMP3, lysosomal-associated membrane protein 3; LC3, light chain 3; NK, natural killer; MHC, major histocompatibility complex; IL, interleukin; IFN, interferons; DM, diabetes mellitus

Statins (HMG-CoA reductase inhibitors) aid in reducing the levels of cholesterol in human macrophages by various mechanisms [5]. Some of these actions include depletion of cholesterol biosynthesis, stimulation of cholesterol efflux as well as inhibition of cholesterol ester accumulation [5]. Therefore, in vitro statin therapy alleviates the process of cholesterol-dependent phagocytosis in macrophages [31]. Apart from lowering cholesterol synthesis in vivo, statins have pleiotropic action [32]. This is the reason how statins prevent the entry of tubercle bacilli in the alveolar macrophages and ultimately, prevent the host from M. TB infection. Apart from this, deficiency of vitamin D3 has been observed to increase the risk of M. TB in various populations [33]. Although statins diminish the levels of cholesterol and are expected to cause deficiency of vitamin D3 which is a downstream product of cholesterol, surprisingly statins either do not alter the levels of vitamin D3 or sometimes cause an increment in its concentration [34-35]. Hence, statins are reported to act as vitamin D3 analogs [36]. This production of vitamin D positively influences pancreatic beta-cell function in a chronic diabetic state, which itself is a known risk factor for M. TB infection due to immunosuppression [37]. It is an established fact that the cholesterol content in the macrophage membrane is relatively higher in diabetics when compared with the normal population [5]. This might be an important factor predisposing diabetics to M. TB infection [9]. Therefore, statins via reducing membrane cholesterol levels can minimize the occurrence of M. TB infection among the diabetic population as well [5]. The anti-tuberculous (anti-TB) effects of statins are summarized in Figure 1. 

What do studies suggest regarding anti-TB effects of statin therapy?

In Vitro Studies

The first study to highlight the potential effects of statins on infection by M. TB was conducted 20 years ago by Montero *et al*., where it was observed that fluvastatin modulates the release of type 1 T helper (Th1) and type 2 T helper (Th2) cytokines and consequently activates caspase-1 or leads to the secretion of interleukin (IL)-1β, IL-18 and interferon gamma (IFNγ) [38]. The peripheral blood mononuclear cells (PBMCs) were observed to be stimulated synergistically when infected with M. TB and treated with fluvastatin. The results suggested that statins could potentiate the host response against M. TB [38]. In 2009, Lu *et al*. reported that lovastatin and fluvastatin interfere with the formation of lipid rafts by inhibiting tyrosine phosphorylation and expression of monosialotetrahexosylganglioside (GM1) and cluster of differentiation 69 (CD69) in gamma delta (γδ) T cells. This leads to inhibition of γδ T cells induced by M. TB antigens [39]. However, both of these studies could not assess the effect of statins on mycobacterial growth and immune response of the host [38-39].

Parihar *et al*. studied the host protective mechanisms in individuals with hypercholesterolemia [12]. The study concluded that PBMCs and monocyte-derived macrophages (MDMs) from patients with familial hypercholesterolemia receiving statin therapy for at least six months were more resistant to mycobacterial infections than the cells of healthy non-statin users. In the same study, bovine bone marrow-derived macrophages (BMDMs) were treated with 50µM of simvastatin and were infected with M. TB for four hours at 37 °C. The results depicted a significant decrease in bacterial growth without alterations in cellular viability. In addition, they also conducted metabolic rescue experiments through confocal microscopy and western blot which demonstrated that statins decrease membrane cholesterol levels and promote phagosomal maturation and autophagy in macrophages. The effect of statins on inhibition of M. TB growth was reversed by phagosome or autophagosome maturation inhibitors like mevalonate. This study suggested the possibility of studying the supplementary role of statins with first-line anti-TB drugs [12].

Lobato *et al*., in 2014, investigated the efficacy of atorvastatin (0.2-2µM) and simvastatin (0.2-2µM) with rifampicin (RIF) (1µg/mL) on the intracellular viability of mycobacteria (*M. leprae* and M. TB) within THP-1 macrophages [40]. After 72 hours, both statins induced bactericidal effects on each strain. With higher doses (2µM atorvastatin and 2µM simvastatin), both drugs reduced the viability of M. TB by about 75%. Additionally, both statins showed an additive effect in combination with RIF (1µg/mL RIF plus 0.2µM atorvastatin plus 0.2µM simvastatin) in case of M. TB infection. However, for *M. leprae* strain, only atorvastatin showed an additional effect with RIF. The mechanism involved in the inhibition of mycobacterial growth was determined by testing the effect of atorvastatin on THP-1 macrophages infected by *M. leprae*. The results concluded that bactericidal effects in the cells infected by *M. leprae* were secondary to phagosomal arrest [40].

To evaluate the tuberculocidal effect of simvastatin alone or in combination with first-line anti-TB drugs, Skerry *et al*. performed an experiment on J774 macrophages [41]. After 2 days of infection, a significant increase in tuberculocidal activity of isoniazid (INH) was observed with exposure to 5µM simvastatin as compared to INH alone. Similarly, simvastatin (25 mg/kg) enhanced bacterial killing when added with the standard oral regimen of RIF (10 mg/kg), INH (10 mg/kg), and pyrazinamide (PZA) (150 mg/kg). This additive bactericidal effect lost statistical significance by day five. Further, it was noted that simvastatin alone lacks anti-TB activity during the acute stage of infection. The study suggested exploring the optimal dose of statin and the ability of combination treatment to accelerate the time required to achieve a stable cure [41]. 

In 2016, Dutta *et al*. studied the adjuvant effect of simvastatin with INH, RIF, and PZA on the duration of corrective treatment [42]. To determine the effects of statins on the activity of first-line anti-TB drugs and intracellular RIF concentration, M. TB-infected THP-1 macrophages were exposed to simvastatin. It was concluded that simvastatin significantly enhances the bactericidal activity of first-line drugs without altering intracellular RIF concentrations. A reduction in the time required to achieve culture-negative lungs from 4.5 to 3.5 months was observed with adjuvant treatment (60 mg/kg simvastatin plus 10 mg/kg INH plus 10 mg/kg RIF plus 150 mg/kg PZA). However, simvastatin did not alter plasma or lung lesion cholesterol levels [42].

Guerra-De-Blas *et al*. analyzed the effects of simvastatin on the treatment of M. TB infection [43]. Direct quantification of M. TB growth was determined using PBMCs infected with M. TB H37Rv at multiplicity of infection (MOI) of 0.1. Although no direct antimicrobial activity was observed, simvastatin decreased the growth of M. TB in PBMCs, increased the proportion of natural killer (NK) T cells in culture and expression of co-stimulatory molecules in monocytes, promoted the secretion of cytokines and activated autophagy in monocytes resulting in a significant depletion in bacterial load. The study also suggested that further research should be conducted to define statin-induced anti-inflammatory mechanisms in TB treatment [43].

To determine whether statins can enhance the activity of anti-TB drugs against intracellular bacilli in macrophages, Dutta *et al*. conducted another study in 2019 [13]. They performed experiments with the M. TB H37Rv and THP-1 macrophages infected at MOI of 20. Pravastatin (7.8 μM), simvastatin (0.2 μM) and fluvastatin (0.032 μM) enhanced the activity of first-line anti-TB drugs (0.006 μM INH plus 0.0055 μM RIF and 81.23 μM PZA). However, atorvastatin and mevastatin showed no effect on mycobacterial growth at non-toxic doses. Among all statins, pravastatin exhibited the most potent adjunctive activity with the least toxicity by modulating phagosomal maturation characteristics in THP-1 macrophages. These observations concluded that pravastatin can be an attractive candidate for host-directed, adjunctive TB therapy [13]. All in vitro studies are summarized in Table 1.

**Table 1:** Summary of in vitro studies regarding anti-tuberculous effects of statin therapy TH1/TH2; helper 1 T/helper 2 T, PBMCs; human peripheral blood mononuclear cells, M. TB.; mycobacterium tuberculosis, IL; interleukin, IFNγ; interferon gamma, γδ; gamma delta, MDMs; monocyte-derived macrophages, MOI; multiplicity of infection, BMDMs; bone marrow-derived macrophages, BCG; bacille calmette guerin, RIF; rifampicin, TB; tuberculosis, INH; isoniazid, PZA; pyrazinamide [12-13], [38-43]

Author (year)	Study type	Objective	Cell type	Antigen strain	Drug name (dosage)	Results
Montero et al. (2000)	In vitro	To study the effects of fluvastatin on TH1/TH2 cytokine release in relation to caspase-1 activation	PBMCs	Heat inactivated M. TB H37Ra (10µg/ml)	Fluvastatin (5µM)	Statins promoted the release of TH1 cytokines (IL-1β and IFNγ) and the activation of caspase-1 enzyme
Lu et al. (2009)	In vitro	To investigate the effect of endogenous cholesterol on lipid rafts formation and activation of γδT cells	PBMCs	M. TB antigen (5µg/ml)	Lovastatin (10µmol/L) or fluvastatin (2µmol/L)	Statins interfered with the formation of lipid rafts and inhibited the activation of γδT cells
Parihar et al. (2014)	In vitro	To study the host-protective mechanism of statins in individuals with familial hypercholesterolemia on statin therapy	PBMCs and MDMs	M. TB H37Rv (MOI 5)	Simvastatin (50µM)	Statin therapy caused a significant reduction in mycobacterial growth, induced immunomodulatory properties in PBMCs and MDMs
Parihar et al. (2014)	In vitro	To study the host-protective mechanism of statins in an experimental murine model	BMDMs	M. TB H37Rv (MOI 5)	Simvastatin (50µM)	Statin therapy caused a significant reduction in mycobacterial growth via promoting phagosomal maturation and autophagy
Lobato et al. (2014)	In vitro	To investigate the efficacy of statins on the intracellular viability of mycobacteria within the macrophage	THP-1 macrophages	M. TB H37Rv (MOI 10), M. bovis BCG (MOI 50)	RIF (1µg/mL) plus atorvastatin or simvastatin (0.2µM)	Statins reduced intracellular mycobacterial viability (by about 75%) and atorvastatin showed an additive effect with RIF
Skerry et al. (2014)	In vitro	To study the tuberculocidal activity of simvastatin alone and in combination with first-line anti-TB drugs	J774 macrophage-like cells	M. TB CDC1551 (MOI 10)	Simvastatin (5µM) plus INH (0.05µg/mL)	Simvastatin significantly increased the tuberculocidal activity of INH at day 3 after infection
Dutta et al. (2016)	In vitro	To investigate whether the addition of simvastatin to the first-line regimen (INH/RIF/PZA) shortens the duration of curative TB treatment	THP-1 macrophages	Bioluminescent M. TB H37Rv (MOI 0.05)	INH (0.011µM), RIF (0.012µM), and PZA (162.5µM) plus simvastatin (0.1µM)	Simvastatin significantly augmented the bactericidal effect of INH, RIF, and PZA alone as well as in combination
Guerra-De-Blas et al. (2019) [43]	In vitro	To analyze the effects of simvastatin on the treatment of M. TB infection	PBMCs	M. TB H37Rv (MOI 0.1)	Simvastatin (1–20µM)	Simvastatin activates several immune mechanisms that favor the containment of M. TB infection
Dutta et al. (2019)	In vitro	To determine whether statins can enhance the activity of anti-TB drugs against intracellular bacilli in macrophages	THP-1 macrophages	Bioluminescent M. TB H37Rv (MOI 20)	INH (0.006μM), RIF (0.0055μM) and PZA (81.23μM) paired with simvastatin (0.2μM), pravastatin (7.8μM) or fluvastatin (0.032μM)	Pravastatin, simvastatin, and fluvastatin enhanced the antitubercular activity of the first-line anti-TB drugs

In Vivo Studies

All in vivo studies depicting the effects of statins on infection by M. TB have been performed on mice. The first study to investigate M. TB infection in a statin-treated experimental model was performed on C57BL/6 mice (8-12 weeks of age) [12]. The mice were treated intraperitoneally with 20 mg/kg of simvastatin or rosuvastatin and phosphate-buffered saline control every two days for six weeks and were infected by low-dose aerosol-based M. TB H37Rv. After monitoring the disease progression, it was concluded that both statins showed a protective response in the infected mice. About 10-fold reduction in bacillary burden was recorded in the spleen, liver, and lungs of mice infected and treated with simvastatin as compared to untreated control models [12]. 

In 2014, Lobato *et al*. evaluated the ability of atorvastatin to potentiate the anti-bacterial effects of RIF [40]. They inoculated 1×10^4^ live *M. leprae *strains into plantar pads of each shepherd model of BALB/c mice. The mice were divided into six groups after one month of infection and were treated for five months. The control groups were not treated. Two other groups were treated with 40 mg/kg and 80 mg/kg of atorvastatin respectively. The other three groups received RIF (10 mg/kg and 1 mg/kg by gavage weekly) alone or a combination of RIF (1 mg/kg) and atorvastatin (80 mg/kg) daily. After six months, it was noticed that atorvastatin reduced bacterial replication and synergized anti-bacterial effects of RIF. Furthermore, no hepatotoxicity or muscle damage was observed with treatment [40].

Subsequently, Dutta *et al*. investigated whether the addition of simvastatin to the first-line anti-TB regimen shortens the duration of curative TB treatment [42]. BALB/c mice, four to six weeks of age were infected by aerosol-based M. TB CDC1551 (3.7 log_10_ CFU). After the progression of infection for about six weeks, the mice were inoculated with RIF (10 mg/kg), INH (10 mg/kg), PZA (25 mg/kg) with and without the addition of simvastatin (25 mg/kg) for five days per week for eight weeks. After four and eight weeks of treatment, a significant reduction in bacterial load was noticed in the mice treated with a combination of simvastatin and standard anti-TB regimen. The number of lung colony-forming unit (CFU) reduced by 1 log_10_ on day 28 and 1.25 log_10_ on day 56. The results suggested that simvastatin can produce complementary anti-bacterial effects when combined with the first-line anti-TB regimen [42].

In 2019, Dutta *et al*. conducted another experiment on female C3HeB/FeJ mice, five to six weeks of age to determine the anti-TB activity of statins in mice [13]. The mice were inoculated by aerosols of M. TB H37Rv calibrated to deliver ~10^2 ^CFU/mouse lung. Six weeks after infection, mice were treated with INH (10 mg/kg), RIF (10 mg/kg), PZA (150 mg/kg) and ethambutol (EMB) (100 mg/kg) supplemented with simvastatin (90 mg/kg), fluvastatin (15 mg/kg) and pravastatin (50 and 90 mg/kg). The treatment was administered once daily for five days a week for eight weeks. Satisfactory results were observed as the adjunctive therapy with statins significantly reduced bacillary burdens in the lungs of mice. The number of lung CFU decreased to 1.28 log_10_, 1.16 log_10_, 0.78 log_10_, and 0.90 log_10_ by adjunctive treatment with simvastatin (90 mg/kg), pravastatin (90 mg/kg), pravastatin (50 mg/kg) and fluvastatin (15 mg/kg), respectively [13]. All in vivo studies are summarized in Table 2.

**Table 2:** Summary of in vivo studies regarding anti-tuberculous effects of statin therapy M. TB; mycobacterium tuberculosis, RIF; rifampin, M. leprae; mycobacterium leprae, INH; isoniazid, PZA; pyrazinamide, TB; tuberculosis, CFU; colony-forming unit, EMB; ethambutol [12-13], [40], [42]

Author (year)	Study type	Objective	Animal model (age)	Antigen strain	Drug name (dosage)	Duration of treatment	Results
Parihar et al. (2014)	In vivo	To investigate M. TB infection in a statin-treated experimental mice model	C57BL/6 mice (8-12 weeks)	Low-dose aerosol-based M. TB H37Rv	Simvastatin or rosuvastatin (20 mg/kg/every other day)	Six weeks	Statins decreased bacilli burden (up to 10-fold) in the infected mice organs along with reduced histopathology
Lobato et al. (2014)	In vivo	Evaluation of the ability of atorvastatin to potentiate RIF’s anti-bacterial effect	BALB/c mice-Shepard’s model	1 × 10^4 ^live M. leprae in 10 μL inoculated in each hind footpad	Atorvastatin (80 mg/kg/day) alone or in combination with RIF (1mg/Kg/week)	Five months	Atorvastatin synergized RIF’s anti-bacterial effect where none of the treatment strategies increased muscle damage or induced hepatotoxicity
Dutta et al. (2016)	In vivo	To investigate whether the addition of simvastatin to the first-line regimen (INH/RIF/PZA) shortens the duration of curative TB treatment	BALB/c mice (4-6 weeks)	Aerosol-based M. TB CDC1551 (3.7 log_10_)	RIF (10 mg/kg), INH (10 mg/Kg) and PZA (25 mg/kg), plus simvastatin (25 mg/kg)	Eight weeks (five days/week)	The combination therapy with simvastatin reinforced mycobacterial killing and reduced the relapse rates when mice were treated for 2.5 and 3.5 months
Dutta et al. (2019)	In vivo	To determine anti-TB activity of statins in mice	Female C3HeB/FeJ mice (5-6 weeks)	Aerosol-based M. TB H37Rv (∼10^2^ CFU/mouse lung)	Simvastatin (90 mg/kg), pravastatin (50, 90 mg/kg) or fluvastatin (15 mg/kg) with INH (10 mg/kg), RIF (10 mg/kg), PZA (150 mg/kg) and EMB (100 mg/kg)	Eight weeks (five days/week)	Statin adjunctive therapy in mice had significantly reduced lung bacillary burdens

Retrospective Studies

Several retrospective cohorts and population-based case-control studies have highlighted the effects of statin on the risk of developing TB. Kang et al. [44], in 2014, evaluated the effects of statin therapy on the development of TB among diabetic patients. It was a retrospective cohort study conducted on patients (aged 20-99 years) with newly diagnosed type 2 diabetes mellitus (DM) based on the South Korean nationwide claims database. Out of 840,899 newly diagnosed type 2 DM patients, 281,842 (33.5%) were statin users. The patients with active TB used less statins than non-TB patients (19.2% with active TB vs. 33.6% without TB). On gauging the potential baseline confounding factors (e.g. age, sex, malignancy, history of silicosis, malabsorption, chronic kidney disease, hypertension, cerebrovascular disease, myocardial infarction, retinopathy, nephropathy or neuropathy), it was concluded that the use of statins was not associated with development of TB in DM patients; adjusted hazard ratio (aHR): 0.98; 95% confidence interval (CI): 0.89-1.07). The authors noticed that although there was considerable development of TB among newly diagnosed type 2 DM patients, the use of statins has no supplementary protective effect on TB incidence [44].

Lee et al. [45] conducted a retrospective cohort study in Taiwan which included 13,981 patients with type 2 DM older than 65 years. The aim of the study was to investigate whether the strong association between TB and DM is independent of the influence of hypertension, dyslipidemia and their associated treatment. To determine the independent effects of DM on the risk of developing TB, Cox proportional hazard regression model was used. After adjusting for age, sex, comorbidities and use of medications, it was observed that statin users altogether had a lower independent association with the risk of developing active TB; risk ratio (RR): 0.76, 95% CI: 0.60-0.97. The study concluded that statins may lower the incidence of active TB infection in aged Taiwanese patients with type 2 DM. However, the level of adherence and dose received by patients is unknown considering that the statin exposure was based entirely on the prescription information compiled from the National Health Insurance Research Database (NHIRD) [45].

Using the database of the Taiwan National Health Insurance Program, Lai et al. [46] conducted a nested case-control study that included patients older than 18 years of age from 1999 to 2011. A total of 8098 newly diagnosed TB cases and 809,800 control patients were included in this study. Statin users were divided into four groups: currents users (patients who were prescribed statins within 30 days before diagnosis of TB), recent users (patients who were prescribed statins within 31 and 90 days before diagnosis of TB), past users (patients who took statin between 91 days and 1 year before diagnosis of TB) and chronic users (patients with summative prescription greater than 90 days). They used three conditional logistic regression (CLR) models to estimate the incident ratio rates (IRRs). It was noticed that all four types of statin users showed a decreased risk of developing active TB. Albeit, the fourth group showed the lowest risk of developing TB (RR: 0.74; 95% CI: 0.63-0.87). Due to the large sample size and use of CLR models along with the inclusion of more than 75 possible confounding factors, this study can be considered as one of the methodologically strong studies [46].

Another population-based case-control study [11] conducted in Taiwan included 8236 subjects newly diagnosed with pulmonary TB from 2000 to 2013 to explore the relation between statin use and pulmonary TB. An equal amount of sex and age-matched subjects without pulmonary TB were randomly selected as control. A multivariate logistic regression (MVLR) model was used to estimate the odds ratio (OR) and 95% CI for pulmonary TB associated with statins use. After adjustment for co-variables, the OR adjusted for pulmonary TB for the subjects who ever used statins was 0.67 (95% CI: 0.59-0.75). In a sub-analysis, the results demonstrated that subjects taking lovastatin had the least probability of developing active pulmonary TB (OR: 0.56, 95% CI: 0.46-0.68). The researchers confirmed that statin use corresponds to a minor but statistically significant reduction in risk of developing TB and that protective effect is stronger for a longer duration of statin use. However, a causal relationship could not be established due to case-control design and lack of information [11].

Su et al. [10] conducted a retrospective nested case-control study from 2000 to 2013 to evaluate the association between statin use and active TB disease. Data from 102,424 statin users (20 years or older) and 202,718 subjects that were age, sex, and enrollment date-matched were analyzed. Statin users included all subjects having a prescription of some type of statin for ≥30 days. Cumulative defined daily dose (cDDD) of statins was calculated and defined by 3 groups: low (<180), medium (180-365) and high (>365). The study revealed a reduced risk of TB among the statin cohort with a hazard ratio (HR) of 0.53 (95% CI: 0.47-0.61; p<0.001). Furthermore, statin use showed a dose-response relationship with the incident of TB disease risk (low: HR 1.06, 95% CI: 0.91-1.24, p=0.477, medium: HR 0.57, 95% CI 0.45-0.72, p<0.001) and high: HR 0.27, 95% CI 0.22-0.33, p<0.001). The study also assessed the covariates identified in previous studies as risk factors for TB disease or comorbidities associated with TB. Despite that, the study suffers from a lack of precision considering that the information regarding the diagnosis of TB and statin use was obtained from the database [10]. 

Subsequently, another retrospective cohort study was performed by Yeh *et al*. to investigate the effects of statins on the risk of developing TB and pneumonia in asthma-chronic pulmonary disease overlap syndrome (ACOS) patients from 2000 to 2010 [47]. Cox proportional regression analysis with time-dependent variables was used to analyze the cumulative TB and pneumonia incidence. After adjusting for multiple confounding factors (e.g. age, sex, comorbidities, medication use), a significantly lower risk of TB with aHR of 0.49 (95% CI: 0.34-0.70] and pneumonia with aHR of 0.52 (95% CI: 0.41-0.65) was observed. Moreover, they calculated aHRs for statins combined with inhaled corticosteroids (ICSs) and oral steroids (OSs). In the case of TB, the aHRs were estimated at about 0.60 (95% CI: 0.31-1.16) and 0.58 (95% CI: 0.40-0.85) for statins combined with ICSs and OSs respectively. While for pneumonia, the aHRs for statins combined with ICSs and OSs were 0.61 (95% CI: 0.39-0.95) and 0.57 (95% CI: 0.45-0.74) respectively. Therefore, the study concluded that there is a lower risk of TB and pneumonia in statin users than non-users regardless of associated risk factors. Subjects using statins combined with ICSs and OSs have a lower risk to develop pneumonia. Similarly, TB risk was lower among users of statins combined with OSs [47].

To examine the association between the use of lipid-lowering agents (LLAs) and outcomes of patients with pulmonary TB receiving anti-TB treatment, Chen *et al*. conducted a retrospective population-based cohort study in patients newly diagnosed with pulmonary TB [48]. Using the Taiwan NHIRD from 2003 to 2010, a total of 1452 adult patients were identified. 5808 matched patients were also selected as control. Among the patients prescribed with LLAs, 1258 people received statin while 295 received fibrate. The incidence of statin and fibrate users was 1.16 and 1.11 respectively (aHR: 1.04, 95% CI: 0.96-1.12) at nine months. Patients who took oral LLAs had a similar incidence of treatment completion at 9, 12, and 24 months as compared to patients who did not take LLAs. The researchers declared no clinical benefit of statins and fibrates over the standard anti-TB regimen. However, further clinical trials to investigate the adjunctive effect of statin and fibrate in the treatment of TB were recommended [48].

The most recently published retrospective population-based study assessed the effects of statins and non-statin LLAs on the risk of TB and herpes zoster among patients with type 2 DM [49]. The participants were divided into three groups: statin users, non-statin users, and LLAs free groups. Time-dependent Cox regression models were used for both statin users and non-users. It was observed that statin users were associated with a lower risk of TB than non-statin users and drug-free groups. Furthermore, high-potency statin users showed a reduced aHR of 0.491 (95% CI: 0.241-0.999) compared to low-potency statin users (aHR: 0.692; 95% CI: 0.455-1.053). Contrarily, altered ratios were observed in the case of herpes zoster. According to this cohort study, statin use was associated with decreased risk of developing TB but a moderately increased risk of developing herpes zoster as compared to non-statin drugs [49]. All retrospective studies are summarized in Table 3.

All studies demonstrating the effects of statins on the risk of developing TB have been conducted in Korea and Taiwan. Hence, verification is required for generalizing results to other populations. In addition, the studies conducted to date are retrospective and contain information from databases, no updated statistics are available to support the findings and a causal relationship cannot be substantiated.

**Table 3:** Summary of retrospective studies regarding anti-tuberculous effects of statin therapy TB; tuberculosis, DM; diabetes mellitus, HTN; hypertension, ACOS; asthma-chronic pulmonary disease overlap syndrome, LLAs; lipid-lowering agents, N/A; not applicable [10-11], [44-49]

Author (year)	Study type	Study duration	Objective	Study participants	Age of participants (in years)	No. of participants	Data analysis	Conclusions
Kang et al. (2014)	Retrospective cohort study	January 1, 2007-December 31, 2010	To evaluate whether statin therapy affects the development of TB among diabetic patients	Newly diagnosed type 2 DM patients who were recently treated with anti-diabetic drugs	20-99	840,894	Cox proportional hazard regression models	TB development was considerable, and statin use was not protective against TB incidence among newly diagnosed diabetics
Lee et al. (2015) [45]	Retrospective cohort study	1998-2009	To investigate whether the strong association between TB and DM is independent of the influence of HTN and dyslipidemia, and its treatment	Taiwanese patients with type 2 DM	More than 65	13,981	Cox proportional hazard regression models	Statin therapy may decrease the incidence of TB infection in elderly Taiwanese patients with type 2 DM
Lai et al. (2016)	Retrospective nested case-control study	1999-2011	To examine whether statin therapy decreases the risk of active TB	New TB cases and control patients	N/A	8098 cases and 809800 controls	Conditional logistic regression models	Statin therapy decreased the risk of active TB where chronic use of statins (>90 days) was associated with the lowest risk
Liao et al. (2017)	Retrospective population-based case-control study	2000-2013	To explore the relationship between statins use and pulmonary TB in Taiwan	Newly diagnosed pulmonary TB patients and sex- and age-matched controls	Equal to or more than 20	8,236 cases and 8,236 controls	Multivariable logistic regression model	Statins are associated with a small but significantly reduced risk of pulmonary TB where the protective effect is stronger with chronic use of statins
Su et al. (2017)	Retrospective nested case-control study	January 1, 2000-December 31, 2013	To evaluate the association between statin use and active TB disease	Statin users without antecedent TB disease and age- and sex-matched non-users	Equal to or more than 20	305,142	Conditional Cox proportional hazards models	Risk of TB was found to be lower among statin users with dose-dependent protection against TB
Yeh et al. (2018)	Retrospective cohort study	2000-2011	To investigate the effects of statins on TB and pneumonia risks in ACOS patients	Statin users and non-users among ACOS patients	Equal to or more than 18	11,256	Cox proportional hazard models with time-dependent exposure covariates	Statin users had lower TB and pneumonia risks
Chen et al. (2019)	Retrospective population-based cohort study	2003-2010	To examine the association between the use of LLAs and outcomes of patients with pulmonary TB receiving anti-TB treatment	Patients newly diagnosed with pulmonary TB and matched individuals	Equal to or more than 20	49,798	Cox regression models	Neither statins nor fibrates provide clinical benefit superior to that achieved with standard anti-TB treatment
Pan et al. (2019)	Retrospective population-based cohort study	2001-2013	To assess the effects of statins vs. non-statin LLAs on the risk of TB and herpes zoster in patients with type 2 DM	Patients diagnosed with type 2 DM taking statins or non-statin LLAs	Equal to or more than 20	49,628	Time-dependent Cox regression models	Statin use was specifically associated with a decreased risk of TB but a moderately increased risk of herpes zoster infection

## Conclusions

In this review, we have gathered all in vivo, in vitro, and retrospective studies highlighting the tuberculocidal effects of statins and their adjunctive effects with first-line anti-TB therapy for the definitive cure of M. TB infection. Studies conducted on in vitro models have shown the immunomodulatory effects of statins and the dominant role of macrophages in resisting M. TB infection in the presence of statins. Moreover, it has been seen that statin therapy promotes phagolysosome maturation as well as aids autophagy. In addition, it has many anti-inflammatory effects, but the knowledge regarding its microbiological effects on the immune system needs to be researched. Studies in mice have shown that statin therapy can decrease the bacillary burden and shorten the duration of anti-TB treatment. However, no information on mechanisms through which statins enhance the anti-microbial effects of these drugs has been provided. We can hypothesize that statins might weaken mycobacterial cell wall making it more vulnerable to the standard TB regimen. In addition, statins may also bolster the immune response of the host and assist in the early eradication of the disease. Many retrospective studies have highlighted the effects of statin therapy on the risk of contracting TB infection. Albeit, these bactericidal effects are less potent than the standard anti-TB therapy. Chronic dose-dependent use of statins (>90 days) has been observed to be associated with further reduction in risk. Since all these researches have been conducted in Taiwan and Korea, the results cannot be generalized to the other populations due to lack of verification. Hence, prospective studies demonstrating the protective effects of statins on active TB should be conducted to identify the causal relationship.
